# Growth, mortality, and exploitation of *Saurida lessepsianus* Russell, Golani & Tikochinski, 2015, from a southern Aegean Sea small-scale fishery: a stock assessment for sustainable fisheries

**DOI:** 10.7717/peerj.19955

**Published:** 2025-08-22

**Authors:** İsmail Reis, Celal Ateş

**Affiliations:** Department of Fishing Technology, Fisheries Faculty, Mugla University, Mugla, Turkey

**Keywords:** Stock assessment, Growth parameters, Lessepsian fish, Saurida lessepsianus, Aegean Sea

## Abstract

This study aimed to determine the stock assessment and growth parameters of *Saurida lessepsianus* in the southern Aegean Sea. The length frequency distribution of the specimens varied between 11.1 and 38.5 cm. It was determined that females, males, and all individuals had positive allometric growth. The estimated growth parameters L, K, t_0,_ and growth performance index (Ø′) were 58.6 cm, 0.141 year −1, −1.01 years, and 2.68, respectively. The reproduction period continued from April to August. The length at which 50% of the fish population reaches sexual maturity (Lm50) for females was calculated as 19.04 cm. The total mortality (Z), natural mortality (M), and fishing mortality (F) of *S. lessepsianus* in the southern Aegean Sea were determined to be 0.39, 0.24, and 0.15, respectively. The exploitation rate (E) was estimated at 0.37. The present biomass was calculated to be 183.24 tons for F_curr_ (0.15). Maximum sustainable yield (MSY) was 820.07 tons, corresponding to F 2.2. It has been concluded that the stocks of *S. lessepsianus* are not overexploited. F_curr_ may be increased to 2/3 (F = 0.6, yield = 546.6 tons) of the fishing level of 2.2 for MSY.

## Introduction

Fish stocks, which constitute a significant part of the biological riches of the Eastern Mediterranean, have been declining due to overfishing problems and other environmental factors. New fish species have migrated from the Red Sea to the Mediterranean through the Suez Canal, which was opened to shorten trade routes between the Mediterranean and the Indian Ocean (Golani, 1998). It has been determined by various researchers that some lessepsian species on the eastern coast of the Mediterranean have extremely high economic value and have made positive contributions to regional fishing activities (Ben-Tuvia, 1973; Spainer & Galil, 1991; Galil, 1994). *Saurida lessepsianus* Russell, Golani & Tikochinski, 2015 is of major commercial importance to the bottom trawl fisheries in the northeastern Mediterranean (Bilecenoglu et al., 2002). Recently, the commercial importance of *S. lessepsianus* has increased and become an economically important fish species in the coast of the southern Aegean Sea small-scale fishery.

*S. lessepsianus*, a member of the family Synodontidae, was previously mistakenly identified as *S. undosquamis* (Richardson, 1848) and *S. macrolepis* Tanaka, 1917 due to their morphological similarities (Russell, Golani & Tikochinski, 2015). The first specimens in the Mediterranean were reported by Ben-Tuvia (1953) as *S. grandisquamis*, a junior synonym of *S. undosquamis*, on the coast of Israel in December 1952. *S. lessepsianus*, widely distributed in the Red Sea, including the Gulf of Suez, is among the Lessepsian species introduced into the Mediterranean *via* the Suez Canal.

*S. lessepsianus* is a benthopelagic piscivorous fish species inhabiting sandy or muddy substrates 20–100 m of depths, but it usually lives at depths from 20 to 30 m (Russell, Golani & Tikochinski, 2015). The species has reached a wide distribution in the eastern Mediterranean from Libya to the southern Aegean Sea, making it one of the most successful colonists of the Levant basin (Russell, Golani & Tikochinski, 2015; Turan et al., 2018). *S. lessepsianus* is among the species prioritized by the General Fisheries Commission for the Mediterranean (GFCM) as part of regional fisheries management efforts. Some researches on the stock assessment, age and growth, fisheries, reproduction and ecology of Lizardfish were studied in the Mediterranean and in the Red Sea (Gucu et al., 1994; Yousif, 2003; El-Greisy, 2005; El-Halfawy, Amal & Ramadan, 2007; Mater, Togulga & Kaya, 1995; El-Etreby et al., 2013; Mahmoud, El-Haweet & Dimech, 2014; Gundogdu & Baylan, 2016). It has also been reported that the economic importance of the species has increased and the landing amount of the species was 115.9 t for the Mediterranean coast of Turkey (FAO, 2024). However, there has been no recent assessment of lizardfish in the coastal waters of the southern Aegean Sea.

Investigation of the age and growth of fish is vital importance for sustainable fisheries management. Age and growth parameters are used to evaluate the population structure and the yield per recruit of fish stocks. Stock assessment is worldwide recognized as an important matter for the sustainable management of aquatic resources. The aim of this research is to decide the latest available information on age and growth, stock assessment, and reproductive period of *S. lessepsianus* for effective management and conservation strategies.

## Materials and Methods

**Figure 1:**
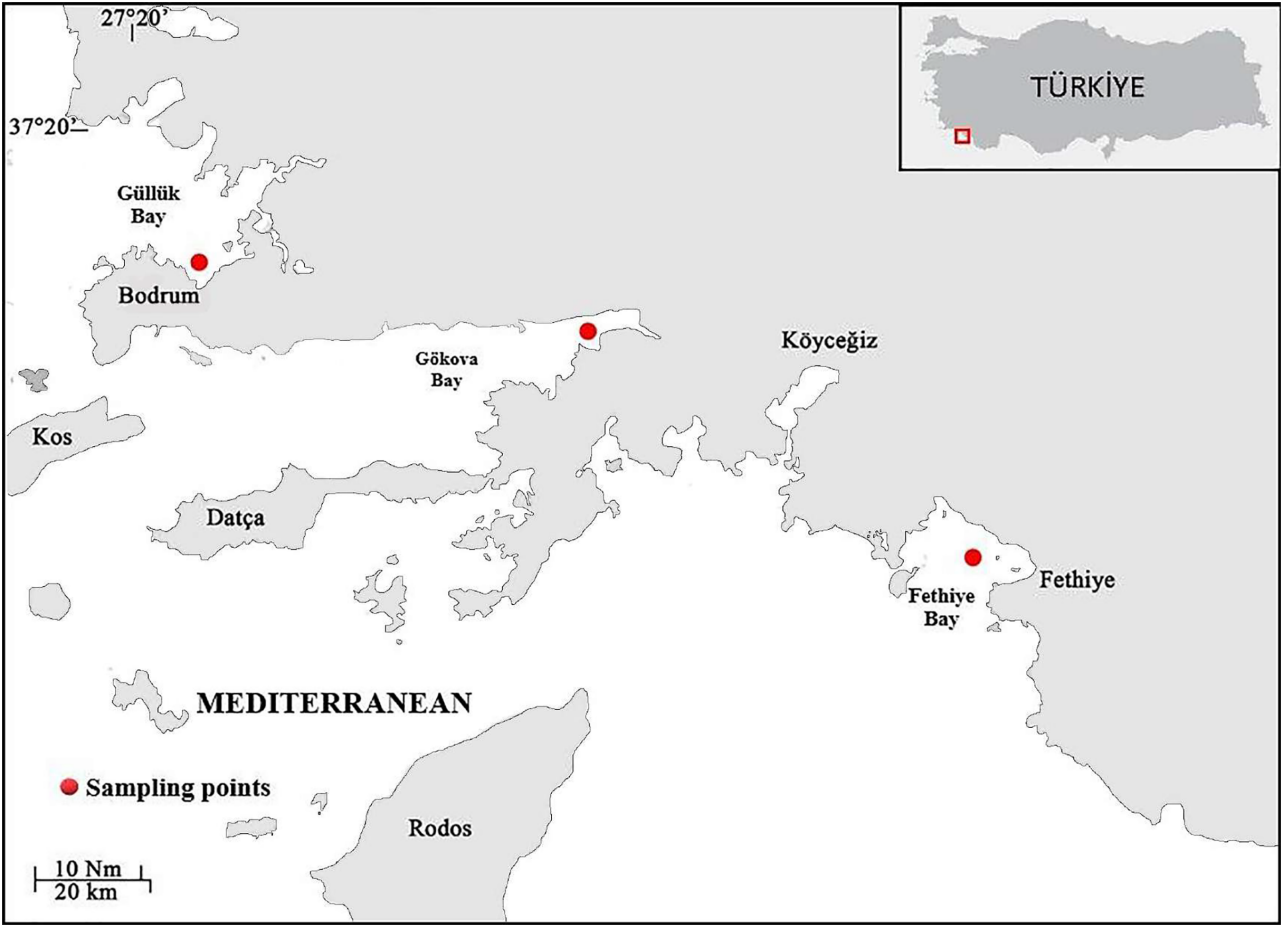
Sampling area (coast of Mugla province in the southern Aegean Sea).

**Figure 2:**
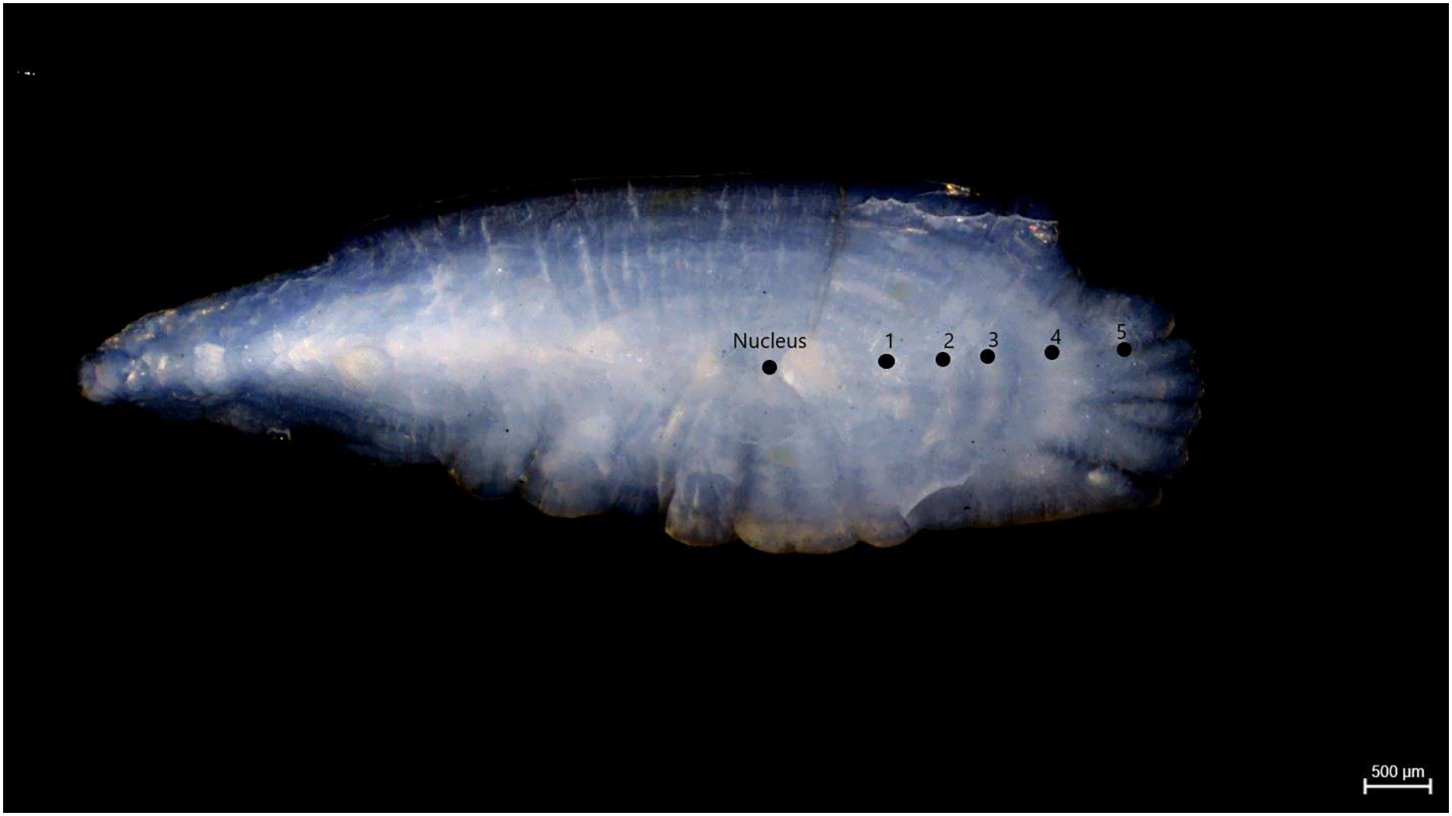
Age rings of S. lessepsianus (TL = 33.6 cm).

Samples of *S. lessepsianus* were caught by commercial fisherman from the coast of Mugla province located in the southwest of Türkiye, Aegean Sea (Fig. 1) between August 2021, and July 2022, using gillnets, were brought to the laboratory, where they then identified using keys provided by Russell, Golani & Tikochinski (2015). Fish total length (TL) and total weight (W) were measured to the nearest 0.1 centimeter and 0.01 gram, respectively. The gill cavity of the fish was opened, and the sagittal otoliths were removed with forceps and cleaned from waste materials. Sagittal otoliths were examined under a stereomicroscope for age determination. The formation of one opaque and one translucent ring was accepted as 1 age (Fig. 2). Age readings were conducted by three independent researchers.

The fish samples used in this study were caught by fishermen. Therefore, no ethics committee certificate was required, and it was approved by the Muğla Sıtkı Koçman University Local Ethics Committee of Aquatic Animal Experiments (Approval No: 2020/11-2).

Sexual identification of all samples was determined by macroscopic examination of the gonads. Gonadal maturity stage of samples was classified according to Brown-Peterson et al. (2011). The sex (♂/♀) ratio of the *S. lessepsianus* was analyzed using Chi Square test (χ^2^) (McHugh, 2013).

The length-weight relationships of the *S. lessepsianus* specimens were represented by the equation W = aL^b^ (Ricker, 1975) was log transformed into the following:

${\rm Iog\;W} = {\rm Iog\;a + b}\;{\rm (Iog\;TL)}.$

The significance of the *b* values for each species was tested by Student’s t-test to confirm that it was significantly different from the predictions for isometric growth (*b* = 3) (Zar, 2010).

Growth parameters were investigated by applying the (Von Bertalanffy, 1938) growth function as follows

${\rm {L_{t}}}= {\rm {L}}_{\infty}\;(1- {\rm e}^{-{\rm K}({\rm t} -{\rm t_0})})$

where Lt is the length at age t, L_∞_ is the asymptotic length, K is the growth coefficient, and t_0_ is the hypothetical age at which length is equal to zero.

The growth performance index (Ø′) was calculated by the equation Ø′ = log K + 2 logL_∞_ (Pauly & Munro, 1984).

The length at which 50% of fish population reaches sexual maturity (L_m50_) was calculated to following formula (King, 1995).

${\rm P} = 1/(1 + {\rm exp}^{\rm {(a-b{^{*}}L)}})$

$${L}_{m50} = - {(a / b)}$$

where P is the maturity percentage of the gonads, *a* and *b* the regression coefficients and L total length of fish.

The gonadosomatic index (GSI) was calculated by the following formula (Avsar, 1998):

${\rm GSI} = {\rm Gonad}\;{\rm weight}/{\rm (Fish\;weight} - {\rm Gonad\;weight}) {^{*}} 100$

The total mortality (Z) was calculated according to Beverton & Holt (1956).

${\rm Z}= {\rm K}^{*}[({\rm L}_{\infty}- {\acute {\rm L}})^{*}({\acute {\rm L}} - {\rm L^{\prime}})]$

where Ĺ is the mean length of fish, and L′ is the length of the first capture.

The natural mortality (M) was calculated using the formula of Pauly (1980).

${\rm logM} = -0.0066 - 0.279\;{\rm log\;L}_{\infty} + 0.6543\;{\rm log\;K} + 0.4634\;{\rm log\;T}$

where T is the annual mean temperature.

The fishing mortality coefficient (F) was estimated from total mortality and natural mortality.

$${\rm F} = {\rm Z}-{\rm M}$$

The exploitation rate (E) was computed from the formula of Gulland (1971).

$${\rm E} = {\rm F/Z}$$

In this research, the relative yield per recruit (Y′/R) and relative biomass per recruit (B′/R) models, developed by Beverton & Holt (1966) and incorporated in FISAT II software (Gayanilo, Sparre & Pauly, 2005), were used to evaluate the stock of *S. lessepsianus*.

${\rm (Y/R)}^{\prime} = {\rm E}^{*}{\rm U^{M/K}}\;[1 - {\rm (3U/1 + m)} + {\rm (3U^{2}/1+2m)} - {\rm (U^{3}/1 + 3m)}]$

${\rm (B/R)}^{\prime} = {\rm (Y/R)}^{\prime}/{\rm F}$

where (Y/R)′ is the relative yield per recruit, (B/R)′ is the relative biomass per recruit, M is the natural mortality coefficient, F is the fishing mortality coefficient, K is the growth parameter, E is the exploitation rate, m = (1−E)/(M/K) = (K/Z), U = 1 − (Lc/L_∞_)

The virtual population analysis (VPA) based on fish length was used to determine the impact of fishing on the stock as well as the MSY by the Thompson and Bell method (Sparre & Venema, 1992).

## Results

**Figure 3:**
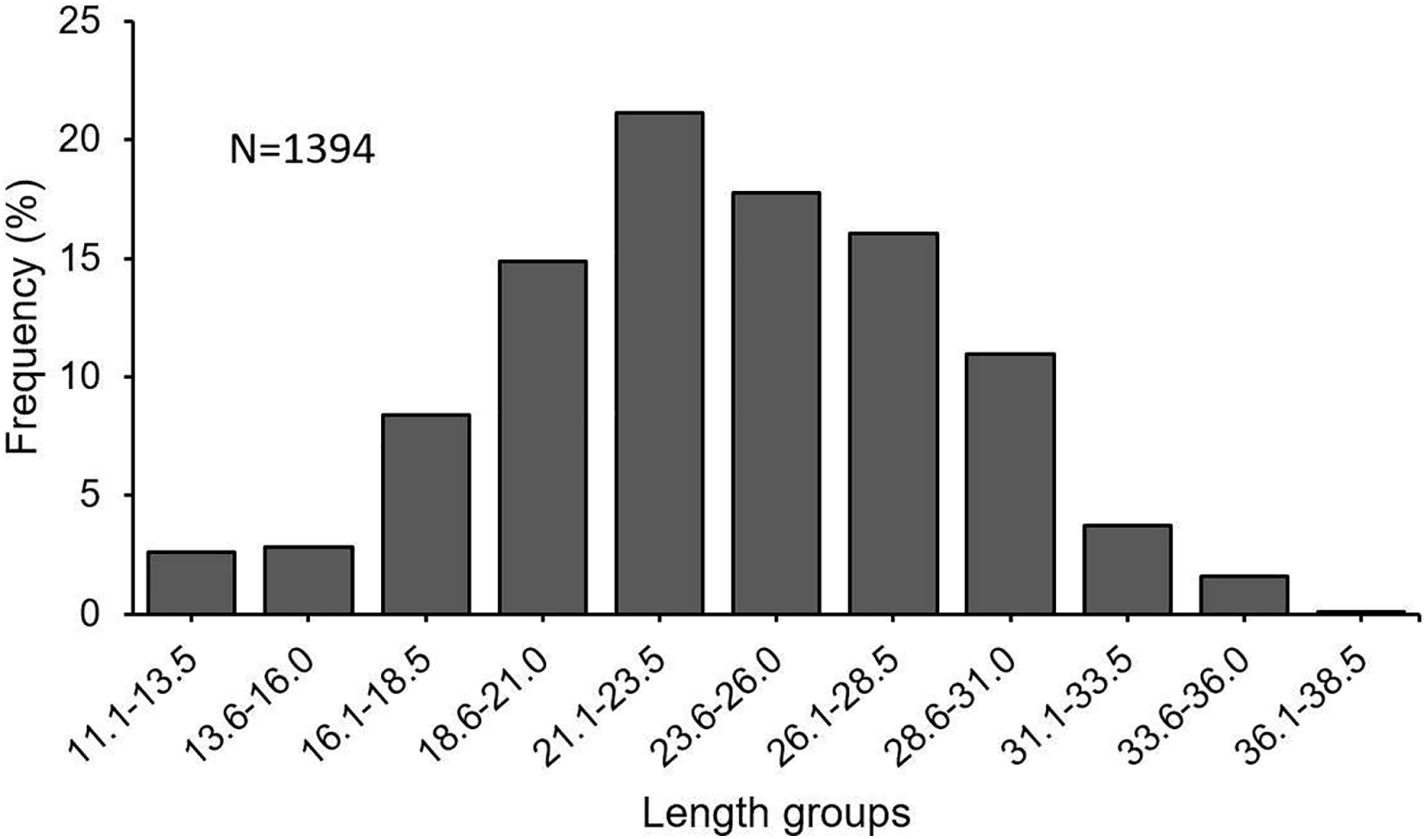
Length–frequency distribution of *S. lessepsianus*, sampled from coast of Mugla province (southern Aegean Sea).

In this investigation, a total of 1,394 specimens (853 females, 482 males, 59 undetermined) were examined. The length frequency distribution of specimens was found to be varying between 11.1 and 38.5 cm (Fig. 3). The fish between 21.0 and 31.0 cm made up 66% of the total catch. The total individual weights were found between 8.49 and 432.86 g. The female: male ratio was estimated at 1 : 0.57. The χ^2^ test revealed that there was a significant difference between the number of females and males (χ^2^ = 103.02; df = 1; *p* < 0.05).

**Figure 4:**
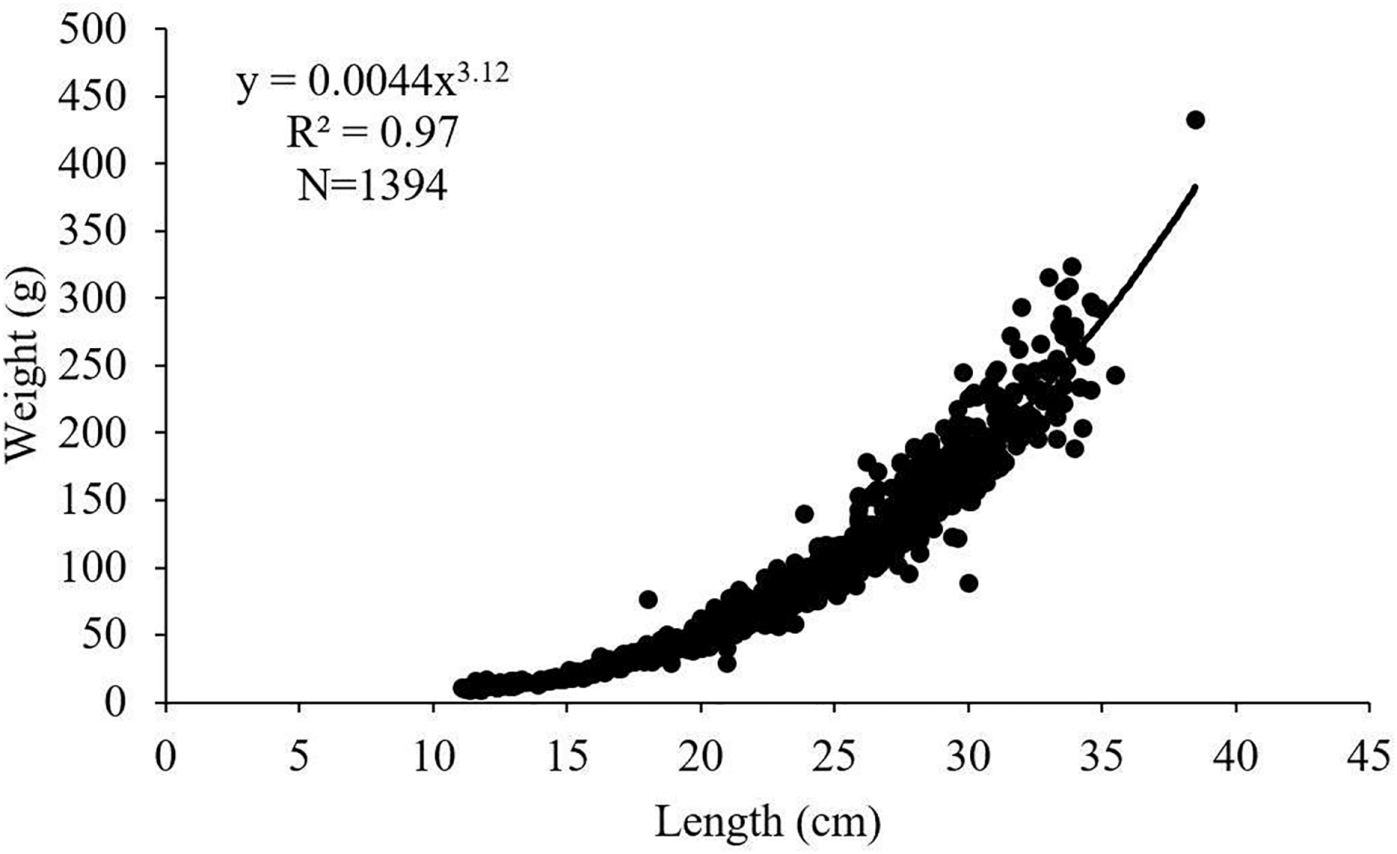
Length–weight relationship of *S. lessepsianus* in the coast of Mugla province (southern Aegean Sea).

The length-weight relationships for female, male and all individuals of *S. lessepsianus* examined in the study were calculated as W = 0.0032 L^3.22^, W = 0.0045 L^3.11^ and W = 0.0044 L^3.12^, respectively (Fig. 4). 95% confidence intervals of b value for females, males and all individuals were 3.18–3.26, 3.05–3.16 and 3.09–3.15, respectively. The calculated b values and the isometric growth value (3.00) were compared with the t-test for females, males and all individuals. It was determined that females, males and all individuals had positive allometric growth (*p* < 0.05).

The specimens of *S. lessepsianus* were represented by five age groups. The most abundant age groups were group II (37.52%) and group III (34.00%), followed by age group IV (18.29%), while age groups I (6.67%) and V (3.52%) had low abundance. Length and weight values corresponding to age groups are presented in Table 1.

**Table 1:** The min., max. and mean length and weight of the different age groups of *S. lessepsianus* in the coast of the southern Aegean Sea.

Ages	N	% Frequency	Length (cm)			Weight (g)		
			min.	max.	Mean	min.	max.	Mean
I	93	6.67	11.1	17.5	14.4 ± 1.79	8.49	31.32	18.44 ± 5.96
II	523	37.52	15.7	23.8	20.4 ± 1.74	19.12	91.31	53.61 ± 14.46
III	474	34.00	22.5	29.4	25.2 ± 1.51	57.11	170.98	103.75 ± 22.57
IV	255	18.29	26.1	33.6	29.4 ± 1.32	95.73	271.78	168.55 ± 30.65
V	49	3.52	31.1	38.5	33.4 ± 1.22	188.05	432.86	251.76 ± 43.94

**Figure 5:**
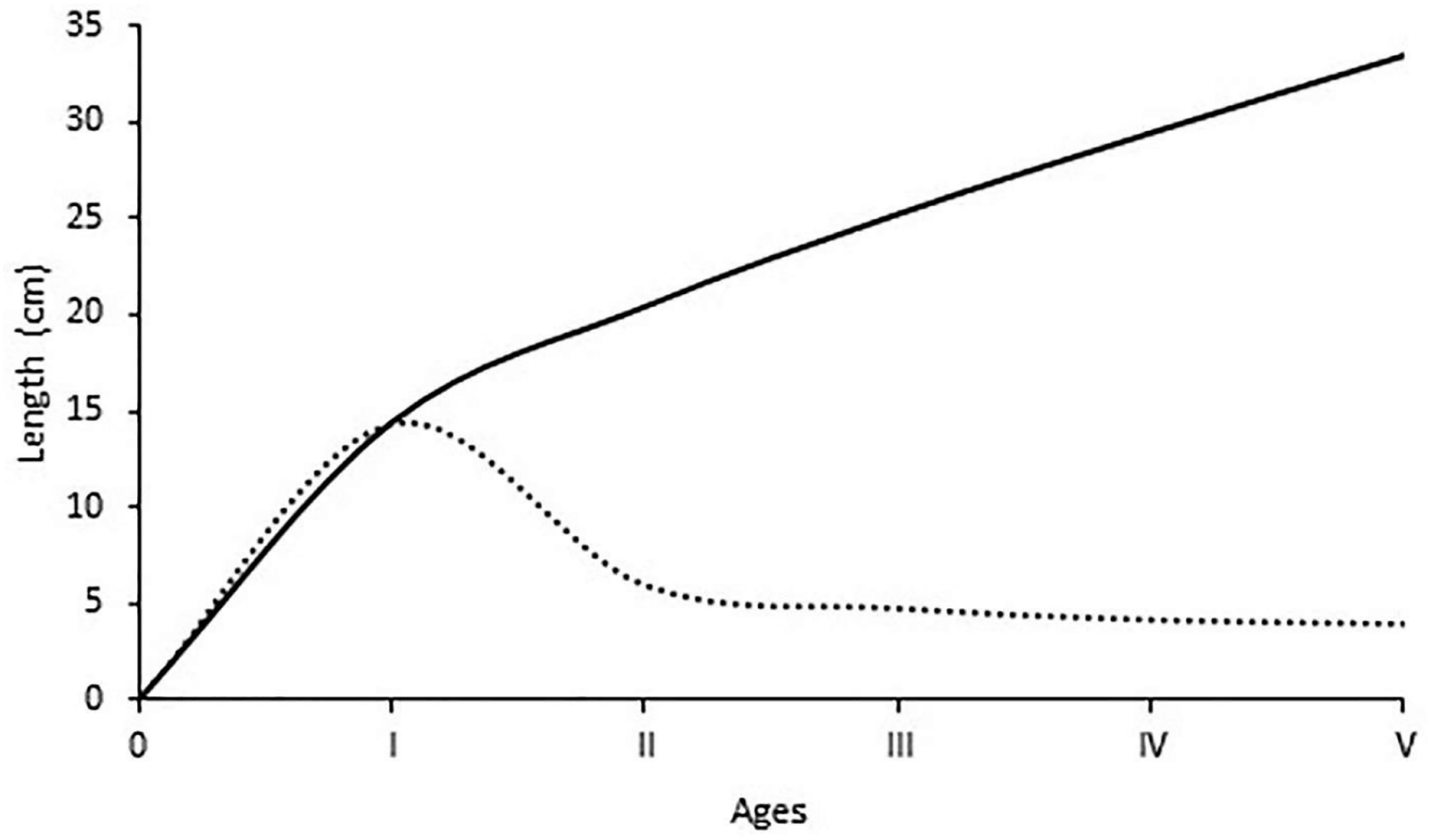
Growth in length and length increments at the end of each age of *S. lessepsianus*.

The average lengths for each age group are given in Fig. 5. The maximum increase in length growth occurred at the end of the first year, and then a gradual decrease in annual length increase occurred with the increment of age. The estimated growth parameters L, K, t_0_ in this study were 58.6 cm, 0.141 year^−1^ and −1.01 years, respectively. The growth performance index was computed as 2.68 for all individuals.

**Figure 6:**
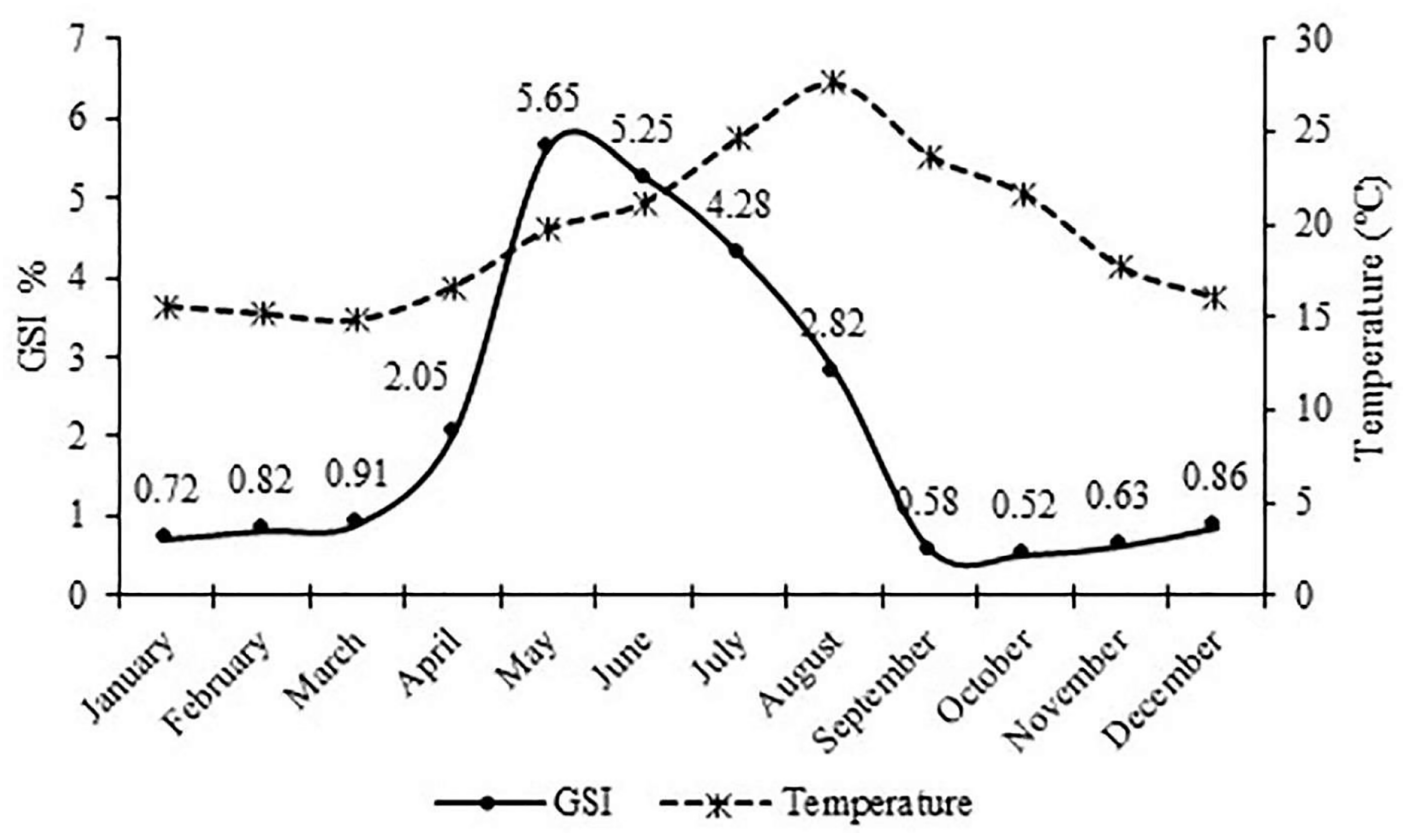
Monthly variation of the gonadosomatic index (GSI) for female *S. lessepsianus* and water temperature on the coast of Mugla province (southern Aegean Sea).

The monthly variations of the gonadosomatic index (GSI) value for females showed that the highest GSI value was found in May with 5.65. The lowest GSI value was found in October with 0.52. Reproduction period continued from early April (16.6 °C) to late August (27.6 °C) with the greatest intensity occurring in May and June (19.7 °C and 21.2 °C) (Fig. 6).

**Figure 7:**
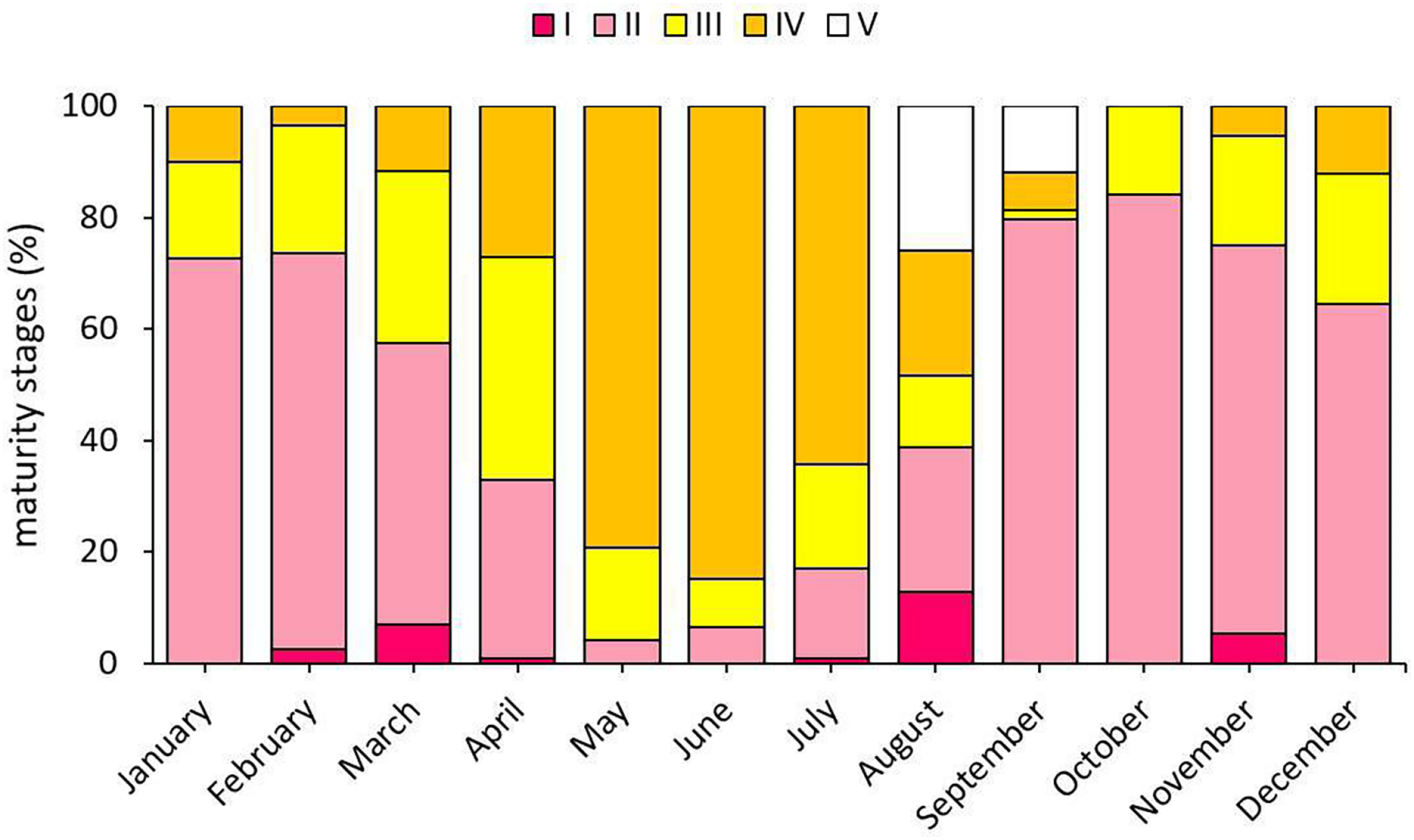
Monthly presence percentages of *S. lessepsianus* individuals according to gonad development.

When the gonad development stages of *S. lessepsianus* individuals were examined, mature individuals were encountered in all months except October. Mature individuals were most intensively detected in May and June, which are within the reproduction season (Fig. 7).

**Figure 8:**
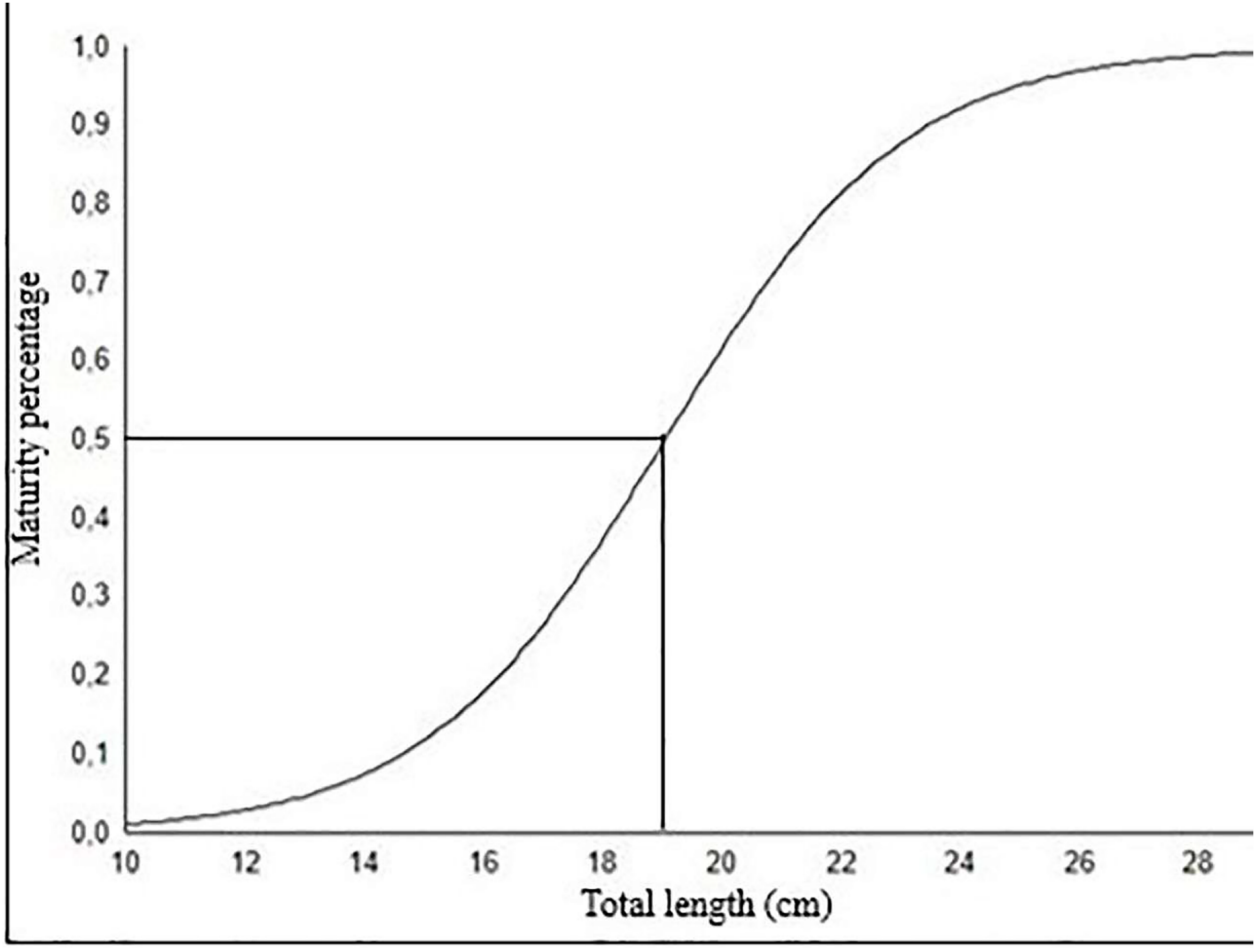
The length at which 50% of fish population reaches sexual maturity (L_50_) for female *S. lessepsianus*.

The length at which 50% of fish population reaches sexual maturity (L_m50_) for females was calculated as 19.04 cm (Fig. 8). This length at first sexual maturity coincides with age group II.

Samples have an average total length of 20.4 cm (15.7–23.8 cm) in age group II.

The total mortality rate, natural mortality rate, and fishing mortality rate of *S. lessepsianus* in southern Aegean Sea Turkish waters were determined to be 0.39, 0.24 and 0.15, respectively. The exploitation rate was estimated at 0.37.

**Figure 9:**
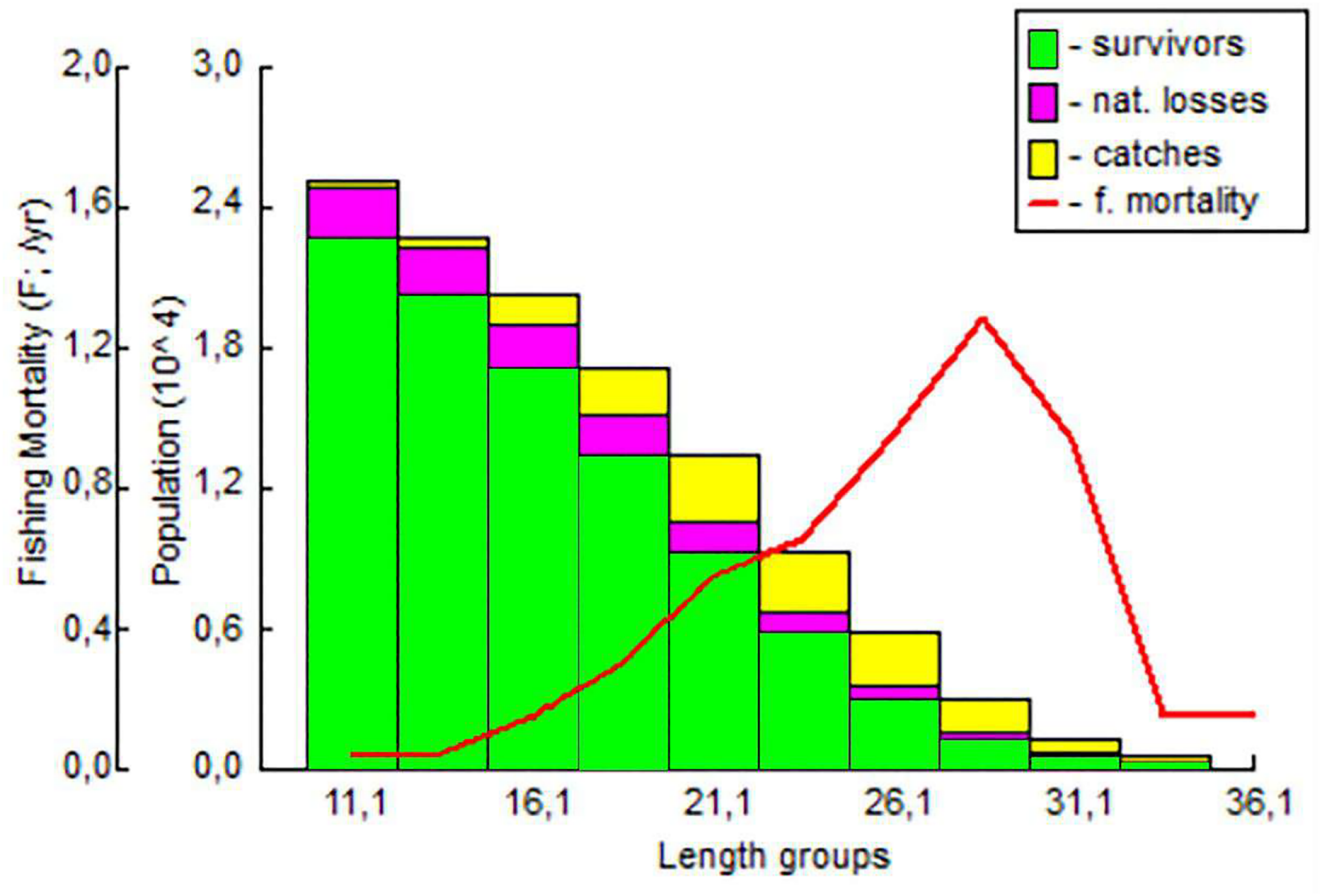
The length-structured VPA for *S. lessepsianus* in the coast of Mugla province (southern Aegean Sea).

It was determined that the fishing pressure on the exploited stock started in the 18 cm length group and peaked in the 28 cm length group. The highest survival rate was determined in the 11.1–16.1 cm length group (Fig. 9).

**Figure 10:**
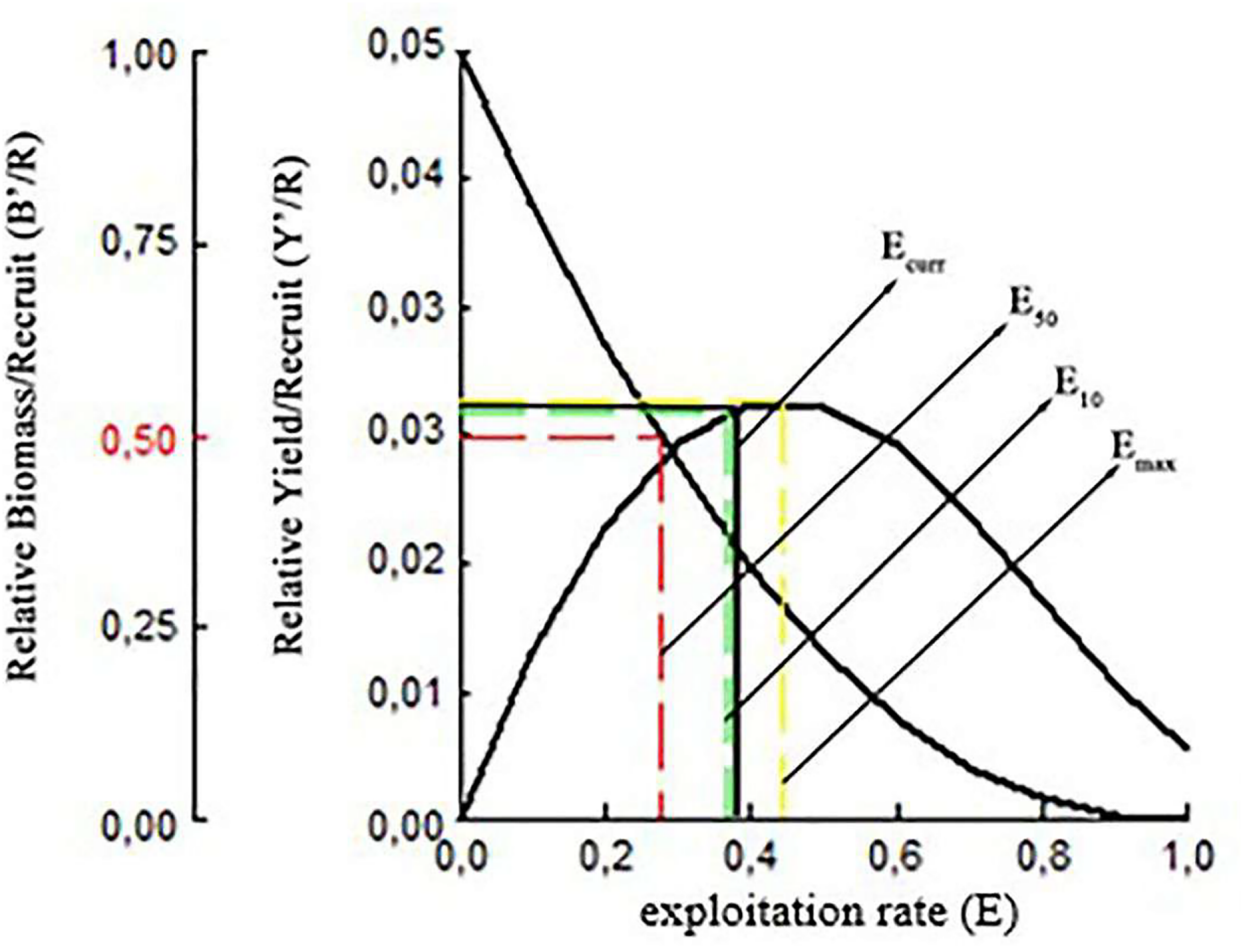
Relative yield per recruit and relative biomass per recruit (B′/R) of *S. lessepsianus* in the coast of Mugla province (southern Aegean Sea).

It was estimated that the maximum sustainable yield (MSY) for *S. lessepsianus* could be achieved at an exploitation rate of E_max_ 0.44 using current fishing techniques. The exploitation levels of E_0.1_ and E_0.5_ were determined 0.36 and 0.28, respectively. Relative yield per recruit(Y′/R) analysis results for *S. lessepsianus* indicate that increased exploitation rate will return some additional catch. The biomass per recruited (B′/R) study findings showed that the biomass per recruited (B′/R) decreased as the exploitation rate increased (Fig. 10).

**Figure 11:**
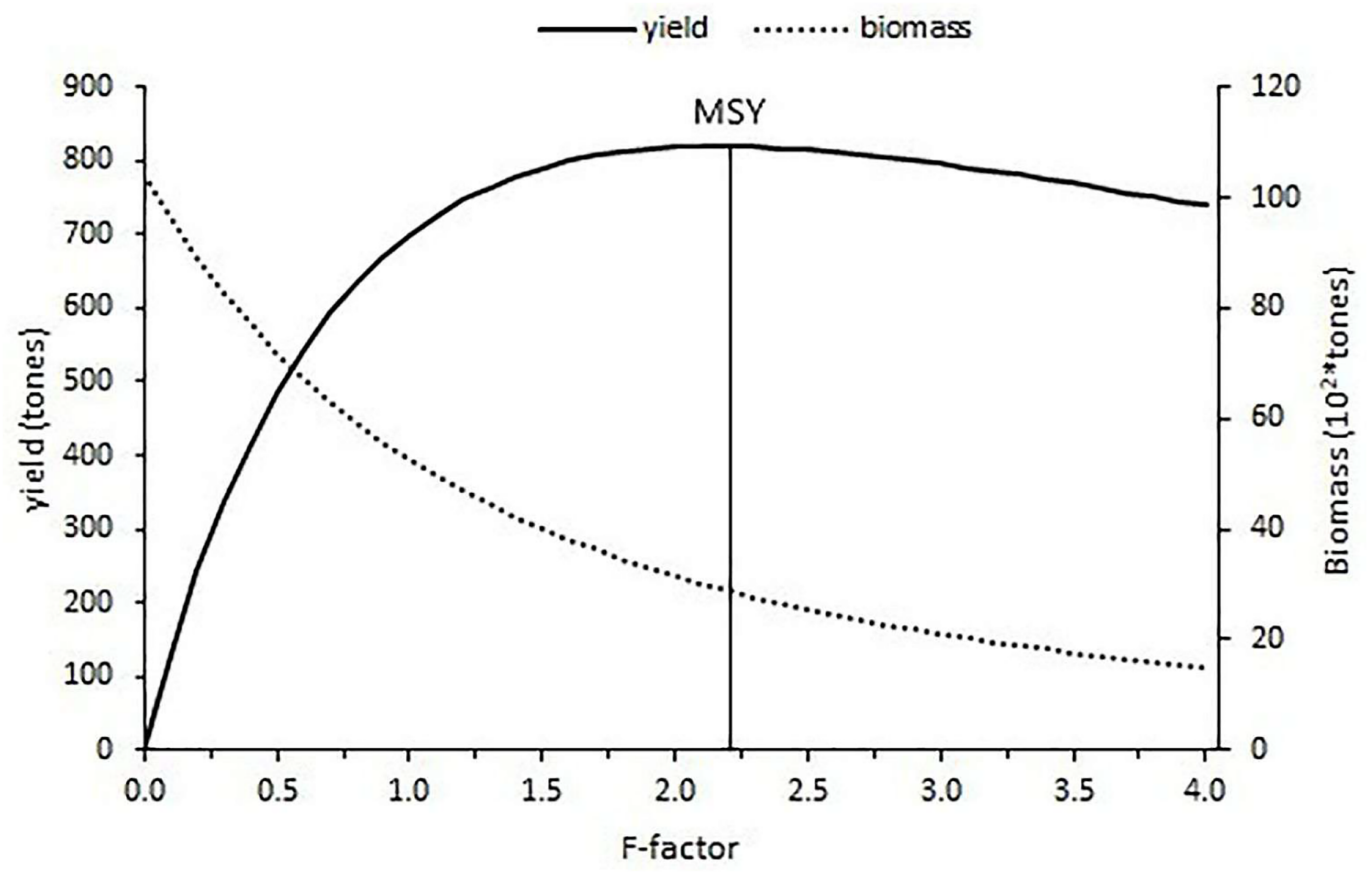
Yield and biomass values at different fishing mortality coefficients according to Thompson and Bell’s prediction.

The current fishing level estimated with an F-factor of 0.15 is calculated as 183.24 tons and the f-factor of 2.2 corresponded to the maximum sustainable yield (MSY) of 820.07 tons with the constant natural mortality (0.24). After the maximum fishing level was reached, the yield gradually decreased for an increase in the fishing level (Fig. 11).

## Discussion

Some lessepsian fish can be commercially important for local fisheries as well as ecological importance. The *S. lessepsianus* has been commercially exploited by the small-scale fishery in the southern Aegean Sea for many years. This study is the first to investigate the age, growth, stock assessment, and reproductive biology of *S. lessepsianus* in the coastal waters of the southern Aegean Sea. In this study, the length distribution of *S. lessepsianus* varies between 11.1 and 38.5 cm. Sangun, Akamca & Akar (2007) give an account of the length range of the species as 10.6–26.1 cm in the Northeastern Mediterranean Coasts. Manasırlı, Avşar & Yeldan (2011) reported the total length of *S. lessepsianus* as 10–21 cm in Babadil Bay. Sulak (1990) stated that the total length of this species is 20–30 cm in the Mediterranean. The length differences between regions may be attributed to variations in sampling time and method.

In the study, the sex ratios were 61.19% for females and 34.58% for males. Similar results were found by other researchers in the eastern Mediterranean (Bingel & Avşar, 1988; Torcu, 1994; Ismen, 2003).

The b value (3.12) of the length-weight relationship of *S. lessepsianus* showed positive allometry (more than ‘‘3’’) *i.e*., the fish becomes heavier for its corresponding length. It is determined that, the b value of this research differs from the various authors in different regions (Can, Basusta & Cekic, 2002; Erguden, Turan & Gurlek, 2009; Cicek & Avşar, 2011; Mahmoud, El-Haweet & Dimech, 2014; Turker, Zengin & Bal, 2020). However, it is close to that presented by Manasırlı, Avşar & Yeldan (2011), El-Etreby et al. (2013), Ozvarol (2014), Yedier, Kontaş & Bostancı (2020), Basusta & Basusta (2022), Uyan et al. (2024). It is estimated that the differences in results between some researchers and this study are due to the sampling method, time, sample size, and the ecological characteristics of the environment in which the species lived.

Von Bertalanffy growth analysis applied on *S. lessepsianus* from the coast of the southern Aegean Sea showed that the species is a relatively fast-growing fish, which is in accordance with findings obtained from the different regions. In contrast to this, it was reported that a very low asymptotic length value and a nonrealistic maximum age, resulting from a sampling strategy that did not include large-sized fish in İskenderun Gulf (Tureli & Erdem, 1997) (Table 2).

**Table 2:** Growth parameter estimates of *S. lessepsianus* from different localities.

L∞ (cm)	K	t_0_	Φ	N	Method	Locality	Authors
22.43	0.597	−1.365	2.47	333	Otolith	İskenderun Bay	Tureli & Erdem (1997) *
42.00	0.178	−1.229	2.50	602	Otolith	İskenderun Bay	Ismen (2002) *
39.5	0.31	−0.31	2.68	1,650	L. frequency	Coast of Visakhapatnam	Rajkumar et al. (2003) *
42.00	0.510	−0.290	2.95	4,711	L. frequency	İskenderun Bay	Gokce et al. (2007) *
41.57	0.118	−1.895	2.31	2,757	Otolith	Babadillimanı Bight	Manasırlı, Avşar & Yeldan (2011) *
51.35	0.131	−1.45	2.54	965	Otolith	Gulf of Suez	El-Etreby et al. (2013) *
41.76	0.232	−0.589	2.61	3,444	L. frequency	Egyptian Medit.	Mahmoud, El-Haweet & Dimech (2014) *
48.86	0.107	−1.733	2.41	400	Otolith	İskenderun Bay	Gundogdu & Baylan (2016)
58.6	0.141	−1.01	2.68	1,394	Otolith	Southern Aegean sea	Present study

**Note:**

* Misidentified as *Saurida undosquamis*.

In the present study, the reproductive season of the *S. lessepsianus*, *i.e*., the period in which the reproductive axis is active, lasts from early April to late August. Kadharsha et al. (2013) stated that *S. lessepsianus* (misidentified as *Saurida undosquamis*) spawn from August to January Southeast coast of India; and in addition to this, Yesilcimen (2015) reported that in İskenderun Bay, *S. lessepsianus* (misidentified as *Saurida undosquamis*) reproduce between May and November. Kolding & Fenchel (1981) was reported that the reproduction period and fecundity of the same species differ from one geographical area to another. Differences in the reproduction period of *S. lessepsianus* from different areas could be due to growth rates, seasonal and ecological conditions, *i.e*., local water temperature and salinity.

Knowledge of the length at first sexual maturity (L_m50_) is important for sustainable fisheries management. It was reported that the first sexual maturity lengths for females were determined as 16.5 cm in İskenderun Bay (Ismen, 2003), and 15.5 cm on the coast of India (El-Etreby et al., 2013). This difference in length at first sexual maturity can be related to differences in environmental factors, especially population densities, water temperature and food availability. In addition, overfishing of large adults with high egg quality and productivity in the stock may also lead to a decrease in the participation to ensure sustainability and a decrease in length at first sexual maturity.

Total mortality, natural mortality, fishing mortality and the exploitation rates of *S. lessepsianus* were compared with other values calculated at different geographic localities (Table 3). It was found that the exploitation rate was higher than 0.5 in different regions, while it was determined 0.37 in this study. In this study, the exploitation rate (E_curr_) estimated for *S. lessepsianus* was lower than Gulland’s optimum exploitation rate of 0.50 (Gulland, 1971). These results show that the stocks of the studied species in different geographical regions have become a target species and are exposed to high exploitation rates, while there is no fishing pressure on the coast of the southern Aegean Sea yet. The reason why there is no fishing pressure on *S. lessepsianus* in the coasts of the southern Aegean Sea can be attributed to the fact that it is caught with small-scale fishing gear and not yet in the bottom trawl catch composition.

**Table 3:** Total mortality (Z), natural mortality (M), fishing mortality (F) and exploitation rate (E) for *S. lessepsianus* in different localities.

Z	M	F	E	N	Locality	Authors
1.07	0.26	0.81	0.76	–	Mersin Bay	Bingel (1987) *
1.81	1.05	0.76	0.58	1,650	India	Rajkumar et al. (2003) *
1.59	0.27	1.32	0.83	390	Gulf of Suez	Amin, EL-Halfawy & Ramadan (2007) *
1.79	0.87	0.92	0.51	4,711	İskenderun Bay	Gokce et al. (2007) *
0.766	0.403	0.363	0.47	2,757	Babadillimanı Bight	Manasırlı, Avşar & Yeldan (2011) *
1.77	0.35	1.42	0.80	279	İskenderun Bay	Cicek & Avşar (2011) *
0.938	0.363	0.575	0.613	3,444	Egyptian Mediterranean	Mahmoud, El-Haweet & Dimech (2014) *
0.39	0.24	0.19	0.37	1,394	Southern Aegean sea	Present study

**Note:**

* Misidentified as *Saurida undosquamis*.

The relative yield per recruit (Y′/R) analysis results have shown that additional fishing effort would provide some additional catch for *S.lessepsianus* in the coast of the southern Aegean Sea, which also means economic return. It could be concluded that *S. lessepsianus* stocks are not in a situation of overexploitation in the coast of the southern Aegean Sea. This means that if fishing level (F-factor) increases from 0.15 per year to 2.2 per year, yield will increase. However, the current fishing level (F-factor = 0.15, yield = 183.24 tons) should be increased to 2/3 (F-factor = 0.6, yield = 546.6 tons) of the fishing level of 2.2 for sustainable fisheries management. However, the suggestions for sustainable fishery management have limitations due to the constant natural mortality rate. The natural mortality rate should be considered.

Some researchers explain the decreases, observed in the stocks of local species or the changes in their habitats (Golani, 1998; Galil & Zenetos, 2002), as the negative ecological effects of lessepsian migration. Hovewer, some researchers attribute the increase in fish production to the settlement of lessepsian species in the region in the Eastern Mediterranean (Mater, Togulga & Kaya, 1995). Therefore, importance should be given to population studies of the lessepsian fish and studies that include their interactions with other species. In terms of the size of the marine environment, a management approach that controls the population of lessepsian species at acceptably low levels is more realistic than eradication (Simberloff, 2021).

## Conclusions

The species makes an economic contribution to local fisheries in the region. With changing climatic conditions, the population of this species will likely move further north and form larger populations in the north. Therefore, knowing the basic population parameters and mortality of the species will contribute to the fisheries management plans that are intended to be made specific to the species in the future.

The coasts of the southern Aegean Sea are one of the important fishing areas of Turkey in terms of ecological and economic aspects. The increase in *S. lessepsianus* fishing in the southern Aegean Sea in recent years shows that the stock of this fish is regeneration. There is still no regular fishing statistics kept in our country for the commercially caught lessepsian fish species and there is no fishing regulation in the legal legislation. Pragmatic solutions should be developed to determine the ecological and socio-economic impacts of the lessepsian species. As a result, sustainable fisheries management policies need to be established and implemented without delay in the coasts of the southern Aegean Sea, where fishing pressure, environmental pollution and tourism activities are intense.

## Supplemental Information

10.7717/peerj.19955/supp-1 Supplemental Information 1 Raw data.

## Additional Information and Declarations

### Data Availability

The following information was supplied regarding data availability:

Raw data is available as a Supplemental File.
